# Efeitos da Imunossupressão em Transplantados Cardíacos por Doença de Chagas

**DOI:** 10.36660/abc.20230798

**Published:** 2024-05-07

**Authors:** Lúcio Moreira Fontenele, Nayara Ribeiro Máximo de Almeida, Breno Rennan de Souza Carvalho, Egidio Bezerra da Silva Neto, Brenda dos Santos Teixeira, Johnnatas Mikael Lopes

**Affiliations:** 1 Universidade Federal do Vale do São Francisco Paulo Afonso BA Brasil Universidade Federal do Vale do São Francisco (UNIVASF), Paulo Afonso, BA – Brasil

**Keywords:** Doença de Chagas, Transplante Cardíaco, Imunossupressão

O acometimento cardíaco na Doença de Chagas (DC) representa a forma mais grave da doença, ocorrendo em 30 a 40 % dos pacientes infectados.^1^ Paciente com insuficiência cardíaca de origem chagásica muitas vezes são refratários ao tratamento medicamentoso, requerendo avaliação de necessidade de transplante cardíaco (TC). Após realização do TC faz-se necessário a imunossupressão do paciente para garantir maior chance de aceitação e vitalidade do enxerto, por outro lado, a imunossupressão é fator contribuinte para recidiva da DC, o que poderia comprometer diretamente o enxerto ou mesmo agravar outros parâmetros de saúde do paciente chagásico, repercutindo diretamente na mortalidade nos próximos 5 anos após transplante.^2^ Muitas das terapias cardíacas implementadas na DC advêm de terapias estudadas em cardiopatias de outras origens, sendo necessário mais pesquisas terapêuticas em populações de indivíduos portadores de DC.^1^

O artigo intitulado *Sobrevida de Pacientes Transplantados Cardíacos com Doença De Chagas Sob Diferentes Regimes de Imunossupressores Antiproliferativos* ^3^ apresenta uma temática de extrema importância para o cenário da atenção aos indivíduos com Doença de Chagas, principalmente no que tange a desfechos danosos como óbito e reativação da doença.

Entretanto, visualizamos uma possibilidade de enriquecer mais as inferências apresentadas pelos autores por meio da distinção entre o conceito de risco para o evento de saúde e o risco de o tempo para o evento ocorrer. Esses dois conceitos são medidos de forma diferente em estudos longitudinais, sendo o risco para o evento medido através do risco relativo ou *odds ratio* e no caso no risco do tempo para ocorrência seria o *hazard ratio* (HR). O RR e o OR medem o risco em um intervalo de tempo fixo e o HR mede a velocidade em que o desfecho acontece.^4^

Dessa forma, o que na verdade os autores pretendiam medir, de acordo com os objetivos apresentados, seria o risco para o óbito e nesta situação a forma correta seria o RR, o qual revela a probabilidade para o óbito mediante alguma característica como a terapêutica imunossupressora. Sendo assim, o que temos apresentado na Tabela 3 é a velocidade (risco) para ocorrência do desfecho óbito.

Queremos sugerir que os autores apresentem uma tabela com análise multivariada por regressão de Poisson evidenciando o RR para o evento óbito assim como a mesma análise para o desfecho reativação da DC, a fim de confirmar a ausência de efeito da variável grupo imunossupressor, aprofundando os achados mostrados na Tabela 2.
