# Supplementary material for: Efeitos da Imunossupressão em Transplantados Cardíacos por Doença de Chagas
Source: Arq Bras Cardiol. 2024 May 7;121(4):e20230798. [Article in Portuguese] doi: 10.36660/abc.20230798 (PMC11081063; doi:10.36660/abc.20230798)
Supplement: Translation

# Translation: Effects of Immunosuppression in Heart Transplant Patients Due to Chagas Disease

**DOI:** 10.36660/abc.20230798i

**Authors:** Lúcio Moreira Fontenele, Nayara Ribeiro Máximo de Almeida, Breno Rennan de Souza Carvalho, Egidio Bezerra da Silva Neto, Brenda dos Santos Teixeira, Johnnatas Mikael Lopes

**Affiliations:** 1 Paulo Afonso BA Brazil Universidade Federal do Vale do São Francisco (UNIVASF), Paulo Afonso, BA – Brazil

**Keywords:** Chagas Disease, Heart Transplantation, Immunosuppresion

Cardiac involvement in Chagas Disease (CD) represents the most severe form of the disease, occurring in 30 to 40% of infected patients.^1^Patients with heart failure of chagasic origin are often refractory to drug treatment, requiring assessment of the need for heart transplantation (CT). After carrying out the CT, it is necessary patient's immunosuppression to ensure a greater chance of graft acceptance and vitality, on the other hand, immunosuppression is a contributing factor to CD relapse, which could directly compromise the graft or even worsen other health parameters of the patient, directly impacting mortality in the next 5 years after transplant.^2^Many of the cardiac therapies implemented in CD come from therapies studied in heart diseases of other origins, requiring more therapeutic research in populations of individuals with CD.^1^

The article entitled Survival of Survival of Heart Transplant Patients with Chagas’ Disease Under Different Antiproliferative Immunosuppressive Regimens^3^presents a theme of extreme importance for the scenario of care for individuals with Chagas, especially concerning harmful outcomes such as death and reactivation of the disease.

However, we see a possibility of further enriching inferences presented by the authors through the distinction between the concept of risk for the event of health and the risk of time for the event to occur. These two concepts are measured in a way different in longitudinal studies, with the risk for the event measured through the relative risk or odds ratio, and in the case of the risk of time to occurrence, it would be the hazard ratio (HR). The RR and the OR measure the risk in a fixed time interval and the HR measures the speed at which the outcome happens.^4^

In this way, what the authors actually intended to measure, according to the objectives presented, would be the risk of death and in this situation, the correct way would be better through RR, which reveals the probability of death based on some characteristic such as immunosuppressive therapy. Therefore, what we have presented in Table 3 is the speed (risk) for the occurrence of the death outcome.

We would like to suggest that the authors present a table with multivariate analysis by Poisson regression showing the RR for the death event as well as the same analysis for the outcome of CD reactivation, to confirm the absence of effect of the group variable immunosuppressant, deepening the findings shown in Table 2.
